# Diet Diversification in *Bombyx mori* Larvae: The Impact of Dandelion on Nutritional and Bioactive Profiles for Targeted Farming Goals

**DOI:** 10.3390/insects16020107

**Published:** 2025-01-22

**Authors:** Aleksandra Trajković, Danka Dragojlović, Gordana Stojanović, Ivana Zlatanović Đaić, Milenko Ristić, Marijana Ilić Milošević, Saša S. Stanković, Vladimir Žikić, Nataša Joković

**Affiliations:** 1 Department of Biology and Ecology, Faculty of Sciences and Mathematics, University of Niš, Višegradska 33, 18000 Niš, Serbia; marijanailic83@yahoo.com (M.I.M.); sasasta@gmail.com (S.S.S.); vladimir.zikic@pmf.edu.rs (V.Ž.); 2 Institute of Food Technology, University of Novi Sad, Bulevar Cara Lazara 1, 21000 Novi Sad, Serbia; danka.dragojlovic@fins.uns.ac.rs; 3 Department of Chemistry, Faculty of Sciences and Mathematics, University of Niš, Višegradska 33, 18000 Niš, Serbia; gordana.stojanovic@pmf.edu.rs (G.S.); ivana.zlatanovic@pmf.edu.rs (I.Z.Đ.); 4 Department of Chemistry, Faculty of Sciences and Mathematics, University of Priština in Kosovska Mitrovica, Lole Ribara 29, 38220 Kosovska Mitrovica, Serbia; milenko.ristic@pr.ac.rs

**Keywords:** bioactive compounds, mulberry leaves, nutritional profile, silkworm, *Taraxacum officinale*

## Abstract

The domesticated silkworm, *Bombyx mori*, reared for sericulture, has gained attention as a promising, sustainable protein source. While studies have focused on pupal powders obtained through natural or artificial mulberry-based diets, this research builds on ethnoentomological knowledge of the use of dandelion as a short-term mulberry substitute, in a mixed mulberry–dandelion diet throughout larval development. Through proximate analyses, amino acid and fatty acid profiling, mineral and carbohydrate composition, as well as antioxidant activity assays, we investigated how dietary variation affects *B. mori* larvae. The mixed diet achieved a distinct nutritional profile, increasing total amino acids to 38.40 g/100 g compared to 32.37 g/100 g in the mulberry-only group, with differences in the content of essential amino acids like isoleucine (2.24 vs. 1.42 g/100 g) and phenylalanine (3.34 vs. 2.82 g/100 g). It also elevated linolenic acid to 74.33% of total fatty acids and introduced sorbitol (13.35 mg/g). Those two compounds were not detected in the larvae reared on a single-plant diet. HPLC-DAD analysis revealed phenolic acids, flavonoids, and riboflavin in both groups, which may serve as potential carriers of antioxidant activity. These preliminary findings demonstrate how dietary modifications can change nutritional profiles and antioxidant properties of *B. mori*. At the same time, they lay the foundation for further research to fully understand diet-induced effects in edible insects.

## Simple Summary

Silkworms have long been reared on mulberry leaves, but this study explores what happens when dandelion is added to their diet. Larvae fed a mix of mulberry and dandelion had higher levels of essential amino acids, such as phenylalanine and isoleucine, along with linolenic acid, an omega-3 fatty acid that was absent in the mulberry-only diet. The mixed diet also contained sorbitol, a health-promoting sugar alcohol, while still provided high protein content. Although silkworms fed exclusively on mulberry leaves showed better antioxidant activity, the dandelion-enriched diet enhanced both fatty acid and amino acid profiles.

## 1. Introduction

The majority of current trends in science and industry, along with their intersection in development centers and startups, can be considered as an intentional alignment with the Sustainable Development Goals (SDGs). These goals, proclaimed by the United Nations in 2015, were adopted by all member states as part of the broader 2030 Agenda for Sustainable Development [1]. Acting as a catalyst, this alignment resulted in a surge to find viable solutions for mitigating systemic crises such as hunger, poverty, and paradoxically, over-consumption [2]. In doing so, it has propelled previously unconventional practices, like insect farming, into the mainstream. Over the past decade, what was once a niche activity in cultures across Africa, Asia, South and Central America, and Oceania has taken a central position in industry investment and academia through its roles in waste management, sustainable food production, and other circular economies. As of 2023, the European edible insect market was valued at approximately USD 1.2 billion [3] and it continues to show growth potential. The rapid acceptance of insect farming is driven largely by its potential for significantly lowering the carbon footprint [4], a critical response to the unsustainable trajectory of traditional livestock farming, both financially and environmentally. Insects are increasingly recognized for their provisioning, regulating, and supporting ecosystem services, with approximately 2000 species, including beetles, caterpillars, bees, wasps, crickets, termites, and ants, recognized globally as edible [5].

Within this context, *Bombyx mori* (L.) holds a prominent position due to its multifaceted economic role [6]. *B. mori* contributes through silk production, a high-value commodity that has retained demand despite the rise of synthetic fibers. Beyond traditional sericulture, it also plays a role in entomophagy, particularly in regions where silkworm pupae are traditionally consumed, and it is gaining traction in Europe as a potential protein source. In recent years, the chemical composition of the silkworm has been discussed from several perspectives, highlighting differences in nutritional profiles depending on its intended use for human consumption [7,8,9,10,11], livestock feed [12,13], or nutraceuticals and supplementation [14,15]. While pupae powder is the primary focus, several studies compare it with larval powders [16,17] for nutritional value. European research also focuses mostly on the silkworm pupae powder [18,19,20,21,22], and Tassoni et al. [23] discussed challenges in comparing nutritional profiles due to underreporting on rearing differences, diet, conditions, hybrids, and strains.

Globally, the silk market is valued at over $10 billion as of 2023, with Asia as the largest producer and consumer, particularly of mulberry silk [24]. Increasing applications with non-silk uses originated mainly in Asia, where byproducts of sericulture (e.g., silkworm pupae) are abundant. Europe’s interest in the species mirrors this trend and is largely research-oriented, centered on sustainable protein sources and bio-based materials, especially in countries with existing sericulture traditions [25]. Initially, *B. mori* was permitted only for non-food animals in the EU, but in November 2021, it was approved for a broader range of farmed animals as part of the EU’s shift toward sustainable, circular feed options. This aligns with the European Food Safety Authority (EFSA) assessment, which states that it provides safe, high-quality nutrients suitable for animal feed [26]. The silkworm is one of the earliest domesticated animals globally, and its core rearing requirements have remained largely unchanged since ancient times. As a monophagous species, silkworms rely on fresh mulberry (*Morus alba*) leaves, making sericulture and moriculture inseparable practices for centuries. Efforts to simplify or optimize silkworm rearing have led to dietary modifications, artificial diets, and advancements in breeding and rearing infrastructure. The key factor that contributes to the success of rearing silkworm larvae is the use of mulberry leaves in their diet, which are not easily available, and their quality varies as the season progresses [23]. Rearing larvae on artificial media has shown limitations, such as reduced yield and altered larval metabolism, but also differences in the nutritional composition of larvae or pupae [18,27].

Our previous study [28] investigated the use of fresh leaves from alternative plants for silkworm rearing and reported the effects of combining mulberry and dandelion leaves on selected biological traits of *B. mori* larvae. This study, centered on non-silk-oriented rearing, examined biological traits such as larval and pupal stage duration, larval mortality, unsuccessful pupation, and cocoon weight. The results confirmed that while dandelion leaves were readily accepted by the larvae, this diet could not fully support their development. However, a mixed diet with a 3:7 mulberry-to-dandelion leaf ratio allowed satisfactory development, and no statistically significant differences were observed in larval weight (approximately 3.7 g on average), larval and pupal duration, or cocoon weight between larvae fed exclusively on mulberry leaves and those fed the mixed diet. Dandelion (*Taraxacum officinale*) leaves are traditionally recognized as a temporary substitute during mulberry leaf shortages [29] and were chosen as a dietary supplement in our study due to their well-documented health benefits for humans and widespread use as a versatile dietary resource [30,31].

This study aimed to examine how this locally sourced dandelion supplementation influenced the nutritional composition of fifth instar larvae of the silkworm, presenting a comprehensive chemical profile for those reared on a mixed-leaves diet compared to a traditional mulberry leaves diet.

## 2. Materials and Methods

### 2.1. Rearing of Larvae and Powder Preparation

Egg clusters of Bombyx mori were obtained from the Research Centre of Agriculture and Environment (CREA-AA) Sericulture Laboratory in Padua, Italy, from a double-cross strain (126 × 125) × (129 × 127), batch no. 1a, produced in June 2022. The larvae were reared in two parallel groups: one fed exclusively on mulberry leaves (M) and the other fed a combination of mulberry and dandelion leaves in a 3:7 ratio (MD), following the protocol described by Trajković et al. [28] (see Supplementary Figures S1–S4). Each feeding group was represented by three replicates, with 100 larvae in each replicate. Newly hatched larvae were reared in a laboratory setting under a 16:8 LD photoperiod, at room temperatures ranging from 19 to 24.5 °C. Fresh leaves were disinfected by first washing them in tap water, followed by dipping them in 2.5% bleach solution for 30 s, then in 70% ethanol for an additional 30 s, and finally rinsing them thoroughly with distilled water. Before feeding, the leaves were cut into strips with widths adjusted to the larval instars (e.g., 0.5 mm for L_1_; whole leaves for L_5_). Early instar larvae were initially reared in high-humidity conditions within perforated Petri dishes, transitioning to larger perforated plastic containers from the second instar onward, but the feeding regimes were consistently maintained from hatching until sampling. No additional food was provided until all leaves were consumed. Upon reaching the third day of the fifth instar, 50 larvae from each replicate were starved for 48 h, then frozen and lyophilized, while the remaining half continued their development. The lyophilized larvae from each replicate group were separately milled into a fine powder, hermetically sealed under vacuum, and stored at −24 °C in airtight containers. Each replicate provided approximately 19.6 g to 21.9 g of larval powder for the subsequent analyses. All analyses were performed in triplicate, with each batch corresponding to one of the feeding group replicates.

### 2.2. Proximate Analysis

Crude protein content was determined using the Kjeldahl method (AOAC 978.04) [32] and 1 g of sample. A standard conversion factor (CF) of 6.25 was applied to estimate protein content from nitrogen levels. This CF is commonly used for analyzing caterpillar powders, which are known to often contain less chitin compared to the pupae or larvae of insects from other orders, such as the frequently analyzed coleopteran larvae [33].

Moisture content was analyzed using AOAC method 934.01, crude fat was measured via Soxhlet extraction using AOAC method 920.39 (5 g of the samples), while ash content was determined using AOAC method 942.05 (3 g of sample) [34]. The results were recalculated and expressed as g/100 g dry weight.

### 2.3. Amino Acid Analysis

Amino acid (AA) analysis was performed on samples by ion-exchange chromatography using an automatic AA analyzer Biochrom 30+ (Biochrom, Cambridge, UK) according to Dragojlović et al. [35]. The technique was based on AA separation using strong cation exchange chromatography, followed by the ninhydrin color reaction and photometric detection at 570 nm and 440 nm (for proline). Samples (50 mg each) were previously hydrolyzed in 0.1% phenol/ 6 M HCl (Merck, Darmstadt, Germany) at 110 °C for 24 h. Essential amino acid tryptophan was not determined because it was destroyed during acid hydrolysis in 6 M HCl. Amino acid content was expressed as g of amino acid in 100 g of sample.

### 2.4. Fatty Acids Composition

Fatty acids from *B. mori* larval powder samples were determined as fatty acid methyl esters (FAMEs), obtained by alkaline transesterification of samples and analyzed by GC-MS [36]. A total of 0.5 g of each sample was dissolved in methanol and added with a stirring to a previously prepared sodium methoxide solution (0.4 g of metallic sodium in 15 mL of anhydrous methanol). This mixture was heated to reflux and quenched with excess ice water. It was then extracted with diethyl ether, and the organic layers were combined, dried with anhydrous magnesium sulfate, and evaporated under reduced pressure. The obtained residue was dissolved in diethyl ether and analyzed by GC-MS.

GC-MS analyses were performed using an HP 6890N gas chromatograph coupled with an HP 5975B mass selective detector (Hewlett-Packard, CA, USA). The gas chromatograph was equipped with a DB-5MS fused silica capillary column (5% phenylmethylsiloxane, 30 m × 0.25 mm, film thickness 0.25 μm). The oven temperature was raised from 70 to 315 °C at a heating rate of 5 °C min^−1^ and then held isothermally for 10 min. The injector and interface were operated at 250 and 320 °C, respectively. The samples, 1 μL of the solution, were injected in a split mode (the flow rate was 1 mL min^−1^). The mass selective detector was operated at the ionization energy of 70 eV, in the 35–650 amu range, with a scanning speed of 0.34 s.

The percentage composition was computed from the GC peak areas without the use of correction factors. Qualitative analysis of samples was based on the comparison of their linear retention indices (relative to n-alkanes on a DB-5MS column) with those from the literature (NIST Chemistry WebBook Database), their mass spectra with those of authentic standards, and GC co-injection with an authentic sample.

### 2.5. HPLC Carbohydrate Analysis

Sample preparation for carbohydrate analysis was performed according to the method previously described by Ji et al. [17], with slight modifications. Five mL of 50% ethanol was added to 1 g of silkworm powder and the samples were vortexed for 20 s. After that, the samples were sonicated for 20 min at 80 °C and then cooled and mixed on a shaker for 15 min at 250 rpm. The samples were then centrifuged for 10 min at 3000 rpm and the resulting supernatants were stored at room temperature until analysis.

Arabinose, cellobiose, deoxyribose, fructose, galactose, glucose, xylose, lactose, maltose, mannitol, melibiose, raffinose, rhamnose, ribose, sucrose, sorbitol, sorbose, and trehalose were used as standards. The initial standard solution was made by dissolving 10 mg of each sugar standard in 1.0 mL of water and then filtered through a 0.45 μm syringe filter. A series of working solutions were prepared by diluting stock solutions two-fold in ranges 0.625–5.00 mg/mL.

Identification and quantification of the silkworm powder sugars were carried out using an HPLC Agilent 1200 Series equipped with an auto-sampler injector, quaternary pump, vacuum degasser, refractive index detector (RID), and ChemStation software (rev. B.04.03, Agilent Technologies, Santa Clara, CA, USA), as previously described [37]. Briefly, 1 mL of filtered sugar extracts or standard solution (by 0.45 μm syringe microfilter) was put into 2 mL HPLC. Twenty μL of samples was injected and elution of the samples was performed in isocratic mode on the ZORBAX (Agilent) NH2 column, 5 μm (4.5 mm × 250 mm). The column was thermostated at 23 °C with a flow rate of 1 mL/min of mobile phase MeCN and water (75:25). The temperature of the RID was 35 °C.

The chromatographic peaks were identified by comparing the retention time of the injected standards solution against the constituents of the sugar extract. Quantification was performed based on calibration curves of each identified sugar.

### 2.6. Mineral Content Analysis

The content of mineral components (Ca, K, Na, Mg, Fe, Mn, Cu, and Zn) in *B. mori* larval powder samples was determined using atomic absorption spectroscopy (AAS) following the SRPS EN ISO 6869:2008 standard [38]. Samples were weighed and carbonized on a hot plate, followed by ashing in a muffle furnace at 550 ± 3 °C. The ash residue was dissolved in 6 M HCl and quantitatively transferred to a 50 mL volumetric flask. Lanthanum nitrate and cesium chloride salts were added as matrix modifiers to ensure accurate measurements. Mineral content was determined using an atomic absorption spectrometer (Varian Spectra AA 10, Varian Techtron Pty Limited, Mulgrave, Victoria, Australia) with an air–acetylene flame. Results were expressed in milligrams of mineral components per kilogram of sample as the means of three replicates.

### 2.7. MDA Content

Malondialdehyde (MDA) content in silkworm powder was determined according to the method of Papastergiadis et al. [39], with some modifications. Extraction of MDA from the samples was performed by adding 2 mL of 7.5% TCA containing 0.1% EDTA to 1 g of the sample. The mixture was homogenized with an Ultraturax (Janke and Kunkel, IKA-Werk, Staufeb, Germany) for 1 min, and 8 mL of 7.5% TCA was added to the homogenate, which was then filtered through 150 mm filter paper. MDA content was measured spectrophotometrically using thiobarbituric acid (TBA). The reaction mixture was made by mixing 2.5 mL of homogenate and 2.5 mL of TBA reagent (46 mM TBA in glacial acetic acid) and then incubated for 35 min at 95 °C. After incubation, the tubes were cooled, and the absorbance of the samples was measured at 532 nm on a UV 1650PC UV-VIS spectrophotometer (Shimadzu Corporation, Tokyo, Japan). Quantification of MDA was performed based on the MDA standard curve, which was prepared at a concentration ranging from 0.6 to 10 μM, and results were expressed in μg/g sample.

### 2.8. GSH Content

Glutathione (GSH) was extracted from the samples using a slightly modified method described by Ye et al. [40]. Two grams of the sample were mixed with 20 mL of water heated to 90 °C, and then the extraction was performed in a water bath at 90 °C for 15 min. The mixture was then immediately cooled on ice and centrifuged at 14,000 rpm for 5 min at 4 °C. The amount of extracted GSH in the supernatant was determined spectrophotometrically using the Ellman reagent [41]. The supernatant (300 µL), 750 µL of 0.2 M Na-phosphate buffer at pH 7.4, and 100 µL of 5,5′-dithio-bis-2-nitrobenzoic acid (DTNB) were mixed and the absorbance was measured at 412 nm. The amount of glutathione was determined using a calibration curve and results were expressed in μg/g sample.

### 2.9. DPPH Assay

The antioxidant potential of the samples was determined by measuring the DPPH radical scavenging capacity of methanol extracts prepared from silkworm powder samples. The methanol extracts were prepared by adding 10 mL of methanol to 1 g of silkworm larval powder and then the mixtures were vortexed and allowed to stand in the dark for 24 h. The extraction was carried out for 1 h using ultrasound. The DPPH assay was performed according to the previously published Blois method [42]. DPPH (2,2-diphenyl-1-picrylhydrazil) dissolved in methanol at a 0.04 mg/mL concentration was used as the reagent. The reaction mixture was prepared by mixing 200 µL of methanol extract with 1800 µL of DPPH solution and kept at room temperature in the dark for 30 min. The absorbance of the solution was then measured at 517 nm. The scavenging activity of DPPH radicals was calculated using the following equation:

Scavenging activity (%) = (A0 − A1) × 100/A0,

where A0 is the absorbance of the starting DPPH solution and A1 is the absorbance of the samples.

### 2.10. HPLC-DAD Analyses

HPLC-DAD analyses were conducted on both the silkworm larval powder samples and the mulberry and dandelion leaves provided to the larvae. The plant material, previously disinfected and prepared in the same manner as it was for larval feeding, was dried at 40 °C and ground into a fine powder, to enable a direct comparison between the nutritional and bioactive profiles of the larval powders (M and MD) and their respective diets. The samples for HPLC-DAD analyses were prepared by adding 5 mL of 50% methanol to 1 g of powder or plant material. The mixtures were vortexed and left overnight at room temperature and further extraction was performed in an ultrasonic bath for 30 min at 50 °C. The samples were centrifuged at 14,000 rpm for 5 min, and the supernatants were filtered through a 0.45 μm syringe filter.

An Agilent Technologies 1100 Series chromatograph equipped with a degasser, a binary pump, a thermostated column (Zorbax Eclipse XDB-C18, 4.6 mm × 150 mm, 5 μm, Agilent Technologies Canada Inc., Mississauga, ON, Canada), and a UV/VIS detector was used for the analysis of flavonoids. The mobile-phase components were 1% aqueous solution of formic acid (HCOOH, A) and acetonitrile (80): H_2_O (15): HCOOH (5%) (D). The mobile-phase gradient was as follows: 0–10 min: 100% A; 10–28 min: 0–25% D; 28–30 min: 25% D; 30–35 min: 25–50% D; 35–40 min: 50–80% D; 40–50 min: 80–0% D. Flow of the mobile phase was 0.8 mL/min, the column was thermostated at 30 °C, and the injected volume was 20 μL. Detection wavelengths were 254, 280, 320, and 360 nm. The identification of the extract components was performed on the basis of retention times and absorption spectra of commercially available standards. Quantification was performed based on calibration curves for gallic acid (peaks at 3.8 and 4.7), caffeic acid (peaks at 20.0, 20.4, 23.4, and 23.8), riboflavin, rutin, and quercetin (quercetin glycosides).

### 2.11. Statistical Analysis

All statistical analyses were performed in Python, using the PyCharm environment (version 2023.2.2), scipy, statsmodels, and pandas. The distribution of each parameter was evaluated using the Shapiro–Wilk test for normality, and for the normally distributed parameters, independent *t*-tests were performed to compare the two feeding groups. The equality of variances assumption was assessed using Levene’s test, and for the parameters where variances were equal, standard independent *t*-tests were applied. For those with unequal variances, Welch’s *t*-test was employed to account for the heterogeneity of variances. For parameters identified as non-normally distributed, the Mann–Whitney U test was used as a non-parametric alternative to compare the groups.

## 3. Results

### 3.1. Protein, Fat, Ash, and Moisture Content

The rearing of silkworm larvae on two different diets resulted in statistically significant differences for ash and fat content (Table 1). The most pronounced variation was observed in ash content, which was significantly higher in the MD sample (7.77 ± 0.22%) compared to the M sample (4.62 ± 0.36%). Additionally, the MD sample exhibited slightly higher moisture and fat content, whereas the M sample contained a greater proportion of crude protein, indicating the influence of diet composition on the nutritional profile of the silkworm powders.

### 3.2. Amino Acid Composition

Diet diversification had a significant effect on the amino acid composition of the larval powder (Table 2). The total amino acid content (TAA) of 38.40 ± 0.43 g/100 g sample was found in the MD sample, which is a higher value compared to the TAA value of 32.37 ± 0.40 g/100 g sample in the M sample. The MD sample also had a higher proportion of essential amino acids and an overall better ratio between essential (EAAs) and non-essential (NEAAs) amino acids.

Sixteen amino acids were found in both samples, and the individual amounts of acids were statistically different between the samples for all amino acids except serine and isoleucine. Phenylalanine was the dominant essential amino acid in both samples with concentrations of 2.82 ± 0.03 g/100 g and 3.34 ± 0.08 g/100 g in the M and MD samples, respectively.

Among the non-essential amino acids, aspartic acid was the most abundant in the MD sample, with a concentration of 4.25 ± 0.04 g/100 g, while the contents of alanine and tyrosine were the highest in the M sample with the values of 3.80 ± 0.03 and 3.74 ± 0.05 g/100 g, respectively. Notably, alanine and tyrosine were the only amino acids present in higher concentrations in the M sample compared to the MD sample. The largest differences between the two samples were observed for glutamic acid, histidine, and isoleucine, which had 1.36, 1.02, and 0.82 g higher contents in the MD sample, respectively. Methionine, histidine, and proline were present in the lowest concentrations in both samples.

### 3.3. Fatty Acid Composition

The fatty acid composition of two tested samples, analyzed by GC-MS and expressed through corresponding fatty acid methyl esters (FAMEs), is presented in Table 3. The five FAMEs identified in samples M and MD represented approximately 99.72 and 99.40% of the total amount of FAMEs detected, respectively.

Unsaturated fatty acids (UFAs) predominated in both samples, accounting for 67.09 ± 0.37% in the M sample and 79.88 ± 0.26% in the MD sample. Among saturated fatty acids (SFAs), palmitic and stearic acids were detected in both samples, with significantly higher levels observed in the M sample. The UFA profiles, however, completely differed between the samples (Table 3). The M sample was dominated by oleic acid (approximately 65.40%), while linolenic acid was the most abundant in the MD sample, comprising approximately 74.33% of the total FAs. Linoleic acid was the only UFA present in both samples, with its content being 3.8 times higher in the MD sample compared to the M sample.

### 3.4. Carbohydrate Composition

The carbohydrate profiles of the silkworm powder samples were distinct, with the total content being 2.5 times higher in the MD sample compared to the M sample (Table 4). Both samples contained deoxyribose, ribose, xylose, fructose, glucose, galactose, and lactose, but mannitol, sorbitol, maltose, and trehalose were exclusively detected in the MD sample. Deoxyribose and ribose concentrations were high in both samples and statistically different, while glucose was present at lower concentration with no statistical difference between the samples.

Sorbitol was the predominant carbohydrate in the MD sample, with a concentration of 13.35 ± 0.56 mg/g, while it was absent in the M sample. Trehalose was also quantified only in the MD sample (0.651 ± 0.12 mg/g). Conversely, lactose was detected at 0.445 ± 0.10 mg/g in the M sample but was below the quantification limit in the MD sample.

### 3.5. Mineral Composition

The metal concentration analysis revealed significant differences between the larval powder samples (Table 5). The M sample showed higher levels of most detected metals, including manganese (8.13 ± 0.23 vs. 3.88 ± 0.18 mg/kg), zinc (71.42 ± 0.21 vs. 27.76 ± 0.30 mg/kg), and iron (19.33 ± 0.23 vs. 12.92 ± 0.26 mg/kg). Magnesium and potassium were also substantially higher in the M sample, at 1159.33 ± 7.63 mg/kg and 15,048.31 ± 1.98 mg/kg, compared to 338.59 ± 55.25 mg/kg and 10,192.59 ± 84.77 mg/kg in the MD sample. Sodium and calcium followed a similar trend, while copper showed the opposite, with higher levels in the MD sample (6.73 ± 2.81 vs. 5.44 ± 0.28 mg/kg).

### 3.6. Quality and Antioxidant Analysis

As part of the quality and safety testing of the samples, the contents of malondialdehyde (MDA) and reduced glutathione (GSH) in the silkworm powders were determined, as well as the DPPH scavenging capability of the sample’s methanol extracts. The values of the three parameters were statistically different for the two samples (Table 6).

The MD sample had higher levels of MDA and GSH than the M sample, with respective contents of 5.30 ± 0.09 μg/g and 54.40 ± 0.86 μg/g. The powder obtained from silkworm larvae reared only on mulberry leaves showed a better ability to scavenge DPPH radicals with an inhibition percentage of 39.56 ± 0.41%.

### 3.7. HPLC-DAD Analysis

Table 7 shows the compounds detected by HPLC-DAD analyses in silkworm larval powders and the plants used in their diets. The analyses revealed 13 components in both silkworm powders that could contribute to the antioxidant activity. All of these compounds, except riboflavin, were found in extracts of mulberry and dandelion leaves. Riboflavin was the most abundant compound in the larval powders, with significantly higher concentrations in the M sample compared to the MD sample. Among phenolic acids found in the silkworm powder samples, two unidentified compounds were quantified using gallic acid as a standard, as the UV spectra of these compounds matched those of gallic acid. These compounds were also detected in plant extracts, but their concentrations were below the limit of quantification. Caffeic, neochlorogenic, and chlorogenic acids, on the other hand, were quantified in plant samples, whereas they were only detected in larval powder samples. Rutin and various quercetin glycosides were the main flavonoids detected in the samples. Different glycosides of quercetin, whose saccharide component was not identified, were quantified in similar concentrations in both larval powder samples. Glycoside 2 was significantly higher in the M sample, whereas glycoside 4 was significantly higher in the MD sample. Rutin was exceptionally abundant in the dandelion sample, but its concentration was below the limit of quantification in silkworm samples.

## 4. Discussion

While previous studies have extensively examined the effects of feeding regimes on metabolic changes during rearing on mulberry leaves or artificial diets [43,44], the presented findings showcase the differences that arise when a mixed diet incorporating two plant species is introduced and the potential for dietary diversification to tailor the nutritional composition of *Bombyx mori.*

Based on our findings from proximate analyses, the mixed diet successfully preserved the characteristically high crude protein content typical of silkworm-derived powders, whether pupal or larval, which is primarily attributed to their mulberry-based diet. The crude protein values obtained in this study are similar to or surpass previously reported values for other fifth instar larval powders [45,46].

The amino acid (AA), fatty acid (FA), and carbohydrate profiles varied significantly between the analyzed powder samples, clearly indicating that the silkworm metabolism was influenced by the diet. The greatest influence of the diet was observed on the composition of fatty acids, with oleic acid being the main component in the M sample and linolenic acid in the MD sample. The high content of unsaturated fatty acids in powders produced from fifth instar larvae, with linolenic acid and oleic acid identified as the dominant fatty acids, has been previously documented [47]. On the other hand, Pongworn [48] marked oleic acid as the major component of the fatty acid profile of silkworm larvae reared on mulberry leaves, which is similar to the results obtained in this study under the same feeding regime. The consumption of foods rich in oleic acid offers numerous health benefits, particularly in supporting cardiovascular health. It has also been shown to play a significant role in the prevention of cancer by modulating oxidative stress and inflammation, as well as in reducing the risk of autoimmune and chronic inflammatory diseases [49]. Oleic acid contributes to improved insulin sensitivity and has neuroprotective properties, making it a key dietary component for preventing metabolic and neurodegenerative disorders [50]. The addition of dandelion to the diet resulted in a significant enhancement of the fatty acid composition in the MD sample, particularly with the increase in linolenic acid, which accounted for approximately 74% of the total fatty acids. This finding is notable as linolenic acid, an essential omega-3 fatty acid, is well-documented for its cardioprotective and neuroprotective properties [51,52]. Such a high proportion of linolenic acid is rarely observed in similar studies. The elevated content is likely derived from the biochemical composition of dandelion, which is known to contain omega-3 fatty acids, particularly alpha-linolenic acid [53]. Studies have shown that dandelion leaves and roots are rich in bioactive compounds, including essential fatty acids, which can be readily metabolized and incorporated into animal tissues [54,55]. This suggests that the silkworm larvae can efficiently utilize these fatty acids and demonstrates the potential of dandelion as a sustainable and low-input dietary supplement for enhancing their nutritional quality, particularly in applications targeting omega-3 enrichment.

Qualitatively, the amino acid profiles were consistent in both samples, with a total of 16 amino acids detected in each. However, the MD sample, derived from larvae reared on the mixed diet, exhibited higher concentrations of most essential and non-essential AAs compared to the M sample. Both powders proved to be good sources of phenylalanine, valine, and lysine, which were the most abundant EAAs in the samples. The intake of phenylalanine via foods naturally rich in this AA or dietary supplements has numerous beneficial effects on human health, including an antidepressant effect and stimulation of melanin synthesis [56]. Foods rich in valine improve insomnia, anxiety, and mitochondrial function, while lysine is an important amino acid for maintaining healthy muscles and bones in humans [57,58,59]. High concentrations of these AAs have already been demonstrated in fifth instar silkworm larvae reared on mulberry leaves [48].

The content of NEAAs varied considerably in the tested samples. Alanine, tyrosine, and aspartic acid were detected in the highest concentrations in the M sample, while aspartic acid, glutamic acid, and alanine were the most abundant amino acids in the MD sample. Glutamic acid, aspartic acid, alanine, and glycine were the most abundant NEAAs in larval powders, as reported by Kweon et al. [60] and You-Young Jo [61]. Aspartic acid, the dominant AA in the MD sample, is an important additive in functional foods and beverages, as it is used together with phenylalanine as aspartame, an artificial sweetener. In addition, diets enriched with aspartic acid influence the strengthening of the immune system, liver detoxification, and improvement of depressive disorders [62]. The powder of larvae fed with mulberry leaves is a good source of alanine and tyrosine. Alanine is used in the food industry to enhance taste, while tyrosine is a precursor of neurotransmitters including epinephrine, norepinephrine, and dopamine, and has a positive effect on the human nervous system and cognitive abilities [63,64].

Carbohydrates also play an important role in contributing to the nutritional value of insect larval powders, with the carbohydrate content in silkworm powders reported to be slightly higher than their fat content [65]. Sorbitol was the main carbohydrate component in the powder derived from mixed-diet-fed larvae. During development, sorbitol and trehalose are the two main sugars in the hemolymph of silkworm larvae, but their concentration typically decreases when the larvae enter the prepupal phase [66]. Since these two carbohydrates were only found in the MD sample, their absence in the M sample aligns with the findings of Trajković et al. [28], which reported a slight delay in pupation in larvae fed a mulberry–dandelion mixed diet. Sorbitol is a sweet sugar alcohol found in various foods used as a low-calorie sweetener, humectant, texturizing agent, or emollient, while some studies also show its prebiotic properties [67]. Both analyzed powder samples exhibited a high concentration of deoxyribose and ribose, sugars that are products of nucleic acid degradation. Ribose is commonly used as an energy supplement due to its beneficial effects on cardiovascular health and its role in alleviating stress-related muscle conditions. However, some studies have reported potential adverse effects associated with excessive ribose intake [68].

The metal concentration analysis demonstrated significant differences in the mineral composition of larval powders obtained from the two dietary groups. The M sample exhibited higher levels of most detected metals, including manganese, zinc, iron, magnesium, potassium, sodium, and calcium, compared to the MD sample. These results align with findings in previous studies that have evaluated mineral profiles of *B.mori* larvae reared on traditional mulberry-based diets, which consistently report high concentrations of essential minerals such as zinc, iron, and magnesium for all developmental stages [17,45]. In contrast, the MD sample exhibited a slightly higher concentration of copper. Copper is an essential trace element critical for enzymatic activity, antioxidant defense, and iron metabolism. However, excessive levels can lead to toxicity and adverse health effects. The elevated copper levels in the MD sample are consistent with studies on dandelion, which has been shown to contain bioavailable copper [69] and efficiently accumulate it from the environment [70].

Further analysis of silkworm larvae powders included the determination of malondialdehyde (MDA) content as a food safety parameter, as well as reduced glutathione (GSH) content, DPPH scavenging activity, and main chemical compounds as indicators of the antioxidant capacity of the samples. MDA is a biomarker of lipid peroxidation in food and its increased content indicates the degree of oxidative damage to food products [71]. An increased level of MDA in products negatively affects the sensory properties of products, but it is also associated with adverse health effects due to the mutagenic potential of this compound [72,73]. The MDA contents of the analyzed samples were 3.09 ± 0.10 and 5.30 ± 0.09 μg/g for M and MD samples, respectively. The higher content of MDA in the powder obtained from larvae on a mixed diet is probably due to a higher content of unsaturated fatty acids and a large amount of linolenic acid, which, as a polyunsaturated fatty acid, is the most subject to oxidative degradation [74]. The obtained MDA values for the analyzed samples are within the limits for animal food products, which are usually 0.1 mg/kg in fresh meat, to more than 10 mg/kg in highly processed or improperly stored products [75].

GSH is a key intracellular antioxidant that protects cells from oxidative damage and its high content in food indicates potential health benefits of food, as well as better nutritional quality and freshness [76]. The GSH content in the MD sample was 54.40 ± 0.86 μg/g, which is a slightly higher value compared to the M sample, which contained 41.87 ± 0.51 μg/g. The GSH level in food of animal origin varies significantly and some approximate values are from 1 to 23 mg of GSH per 100 g [77].

The antioxidant capacity of the samples was evaluated using the DPPH method, which determined the ability of the methanol extract prepared from the powder samples to scavenge the DPPH radical. The M sample showed better scavenging activity of DPPH radicals. The antioxidant activity of silkworm powders is highly dependent on the silkworm strains used to produce the powder. Wannee and Luchai [78] reported that the DPPH scavenging activity of extracts prepared from powders of five different fifth-larval-stage strains ranged from 11.95 to 58.96%, which is consistent with the results of this study. Analysis of the phenolic composition in the samples, evaluated as potential carriers of antiradical activity, revealed no significant differences in either the profiles or concentrations of phenolic compounds between the two samples. Unidentified phenolic acids and quercetin glycosides were the major phenolic compounds, while caffeic acid, neochlorogenic acid, chlorogenic acid, and rutin were detected below the limit of quantification in both samples. Quercetin, astragalin, isoquercetin, rutin, and kaempferol were found to be the major flavonoids in steamed and freeze-dried silkworm powder, while quercetin, (+)-catechin, (−) epicatechin, and naringenin were the major carriers of antioxidant activity in powders of five different silkworm strains [78,79].

To determine the origin of the phenolic compounds in the larvae, HPLC-DAD analysis of methanol extracts from mulberry and dandelion was performed. All phenolic compounds detected in the powders were also found in the plants used in the diet, although the concentrations of these compounds were different compared to the powders. Bioactive components in insects originate from the plants they feed on but can also be modified or synthesized de novo in the insects themselves [80]. Thus, riboflavin was detected only in the larval powder samples, while it was absent in the plants. A significantly higher amount of riboflavin was detected in the M sample, which may have contributed to its overall better antioxidant activity. Riboflavin exhibits its antioxidant potential by enhancing the activity of antioxidant enzymes within cells, directly neutralizing free radicals, and influencing the levels of other key antioxidants, such as vitamin C and vitamin E [81]. Both samples demonstrated a high concentration of riboflavin, which suggests that they can serve as valuable dietary sources of this essential vitamin. HPLC-DAD findings suggest dietary supplementation with dandelion alters the chemical composition of silkworm larvae, while compounds abundant in plants, such as rutin, remain below quantification in the larval powders.

Our findings demonstrate that incorporating dandelion leaves into the silkworm diet offers economic and nutritional benefits suitable for edible insect applications rather than traditional silk production. Dandelion is a low-input plant that grows prolifically across Europe, thriving in diverse environments without requiring significant agricultural resources. This makes it an economically viable alternative to mulberry, particularly in regions where mulberry cultivation has declined. It is already cultivated for various uses, such as herbal teas and supplements, but the possible expansion of the naturally sourced rubber industry [82] could lead to an oversupply of dandelion biomass. In such a scenario, utilizing surplus dandelion as a feedstock for silkworm could add value to a potentially underutilized resource. Also, the results contribute to SDG 2 (Zero Hunger) by highlighting the potential of silkworm larvae as a sustainable, high-quality protein source, and to SDG 12 (Responsible Consumption and Production) by demonstrating the feasibility of incorporating low-input plants into insect diets to enhance their nutritional profiles. The long-term effects of the combined-leaves diet on traditional uses remain beyond the scope of this study and should be addressed in future research.

The presented study also implies the broader need for metabolic studies exploring how different plants influence insect composition. Current research predominantly focuses on substrates like agricultural waste or artificial diets, which can create a trade-off between cost-effectiveness and the nuanced impact of high-quality natural diets. With further research, encompassing diverse hybrids and strains, as well as consideration of larval sampling timing, *B. mori* has significant potential to serve markets focused on functional benefits and sustainable sourcing.

## Figures and Tables

**Table 1:** Proximate composition (%) of the samples.

Parameters	Samples	
	**M**	**MD**
Moisture content	9.26 ± 0.24	9.94 ± 0.30
Ash content	4.62 ± 0.36 ^a^	7.77 ± 0.22 ^b^
Crude protein content	73.65 ± 0.28	68.46 ± 0.27
Fat content	9.30 ± 0.11 ^a^	9.66 ± 0.09 ^b^

The results are presented as mean ± SD. Different lowercase superscripts indicate *t*-test significant differences (*p* < 0.05) within the same row. The lower value is always marked as ‘a’.

**Table 2:** Amino acid profiles of the samples.

Amino Acids	Content (g/100 g Sample)	
	M	MD
Essential amino acids (EAAs)		
Valine	2.33 ± 0.02 ^a^	2.51 ± 0.02 ^b^
Methionine	0.86 ± 0.02 ^a^	1.00 ± 0.02 ^b^
Isoleucine	1.42 ± 0.14	2.24 ± 0.02
Leucine	1.76 ± 0.04 ^a^	2.40 ± 0.02 ^b^
Phenylalanine	2.82 ± 0.03 ^a^	3.34 ± 0.08 ^b^
Histidine	0.13 ± 0.03 ^a^	1.15 ± 0.01 ^b^
Threonine	1.59 ± 0.05 ^a^	2.08 ± 0.04 ^b^
Lysine	1.98 ± 0.06 ^a^	2.49 ± 0.06 ^b^
Non-essential amino acids (NEAAs)		
Aspartic acid	3.63 ± 0.10 ^a^	4.25 ± 0.04 ^b^
Serine	2.56 ± 0.02	2.53 ± 0.04
Glutamic acid	1.98 ± 0.05 ^a^	3.34 ± 0.02 ^b^
Proline	0.24 ± 0.01 ^a^	0.73 ± 0.11 ^b^
Arginine	2.10 ± 0.04 ^a^	2.65 ± 0.03 ^b^
Tyrosine	3.74 ± 0.05 ^b^	2.97 ± 0.05 ^a^
Glycine	1.28 ± 0.03 ^a^	1.68 ± 0.09 ^b^
Alanine	3.80 ± 0.03 ^b^	3.10 ± 0.02 ^a^
Cystine	nd	nd
Total amino acids (TAAs)	32.37 ± 0.40 ^a^	38.40 ± 0.43 ^b^
Total EAAs	12.91 ± 0.02 ^a^	17.22 ± 0.15 ^b^
Total NEAAs	19.34 ± 0.14 ^a^	21.25 ± 0.20 ^b^
EAAs/NEAAs	0.668 ± 0.005 ^a^	0.810 ± 0.002 ^b^

nd: not detected. The results are presented as mean ± SD. Different lowercase superscripts indicate *t*-test (Val, Met, Phe, Thr, Lys, Asp, Pro, Arg, Tyr, Gly, Total NEAAs) or Mann–Whitney U test (Leu, His, Glu, Ala, TAAs, EAAs/NEAAs) significant differences (*p* < 0.05) within the same row.

**Table 3:** Fatty acid composition of the samples.

RI	Compound	Designation	Class	Content (%)	
				M	MD
1927	Methyl palmitate (methyl hexadecanoate)	16:0	SFAME	18.89 ± 0.49 ^b^	10.98 ± 0.30 ^a^
2096	Methyl linoleate (methyl (9Z,12Z)-9,12-octadecadienoate)	18:2	UFAME	1.69 ± 0.19 ^a^	5.55 ± 0.20 ^b^
2106	Methyl oleate (methyl (Z)-9-octadecenoate)	18:1	UFAME	65.40 ± 0.32	nd
2115	Alpha-linolenic acid methyl ester (methyl (9Z,12Z,15Z)-9,12,15-octadecatrienoate)	18:3	UFAME	nd	74.33 ± 0.17
2126	Methyl stearate (methyl octadecanoate)	18:0	SFAME	13.74 ± 0.66 ^b^	8.54 ± 0.40 ^a^
2332	Arachidic acid methyl ester (methyl eicosanoate)	20:0	SFAME	tr	tr
	Total			99.72 ± 0.90	99.40 ± 0.56
	Saturated fatty acid methyl esters (SFAMEs)			32.63 ± 0.82 ^b^	19.52 ± 0.50 ^a^
	Unsaturated fatty acid methyl esters (UFAMEs)			67.09 ± 0.37 ^a^	79.88 ± 0.26 ^b^

SFA: saturated fatty acid, UFA: unsaturated fatty acid, tr: trace (<0.05%), nd: not detected; different lowercase superscripts indicate Mann–Whitney U test significant differences (*p* < 0.05) within the same row.

**Table 4:** Carbohydrate profiles in analyzed samples.

RT	Detected Sugars	Concentration (mg/g)	
		M	MD
5.46	Deoxyribose	7.58 ± 0.30 ^a^	8.18 ± 0.18 ^b^
	LD 0.304		
	LQ 1.152		
7.039	Ribose	2.17 ± 0.24 ^a^	3.74 ± 0.41 ^b^
	LD 0.040		
	LQ 0.134		
7.165	Xylose	+	+
	LD 0.016		
	LQ 0.055		
8.485	Fructose	+	+
	LD 0.030		
	LQ 0.103		
9.50	Glucose	0.27 ± 0.07	0.35 ± 0.04
	LD 0.010		
	LQ 0.033		
9.168	Galactose	+	+
	LD 0.771		
	LQ 2.572		
10.022	Mannitol	-	+
	LD 0.030		
	LQ 0.100		
10.478	Sorbitol	-	13.35 ± 0.56
	LD 0.257		
	LQ 0.859		
12.015	Sucrose	+	+
	LD 0.188		
	LQ 0.626		
14.471	Maltose	-	+
	LD 0.567		
	LQ 1.892		
15.524	Trehalose	-	0.651 ± 0.12
	LD 0.023		
	LQ 0.076		
15.554	Lactose	0.445 ± 0.10	+
	LD 0.125		
	LQ 0.416		
Total		10.465 ± 0.83 ^a^	26.271 ± 0.96 ^b^

RT: retention time of sugar standards, LD: limit of detection, LQ: limit of quantification, -: below the detection limit, +: below the quantification limit. The results are presented as mean ± SD. Different lowercase superscripts indicate *t*-test (deoxyribose, ribose) or Mann–Whitney U test (total) significant differences (*p* < 0.05) within the same row.

**Table 5:** Mineral composition of larval powder samples.

Detected Metals	Concentration (mg/kg)	
	M	MD
Mn	8.13 ± 0.23 ^b^	3.88 ± 0.18 ^a^
Zn	71.42 ± 0.21 ^b^	27.76 ± 0.30 ^a^
Fe	19.33 ± 0.23 ^b^	12.92 ± 0.26 ^a^
Mg	1159.33 ± 7.63 ^b^	338.59 ± 55.25 ^a^
K	15,048.31 ± 1.98 ^b^	10,192.59 ± 84.77 ^a^
Na	1214.49 ± 0.53 ^b^	468.77 ± 8.92 ^a^
Ca	1008.64 ± 3.21 ^b^	342.14 ± 7.87 ^a^
Cu	5.44 ± 0.28 ^a^	6.73 ± 2.81 ^b^

The results are presented as mean ± SD; different lowercase superscripts indicate Mann-Whitney U test significant differences (*p* < 0.05) within the same row.

**Table 6:** MDA, GSH content, and DPPH activity.

Parameters	Samples	
	M	MD
MDA (μg/g)	3.09 ± 0.10 ^a^	5.30 ± 0.09 ^b^
GSH (μg/g)	41.87 ± 0.51 ^a^	54.40 ± 0.86 ^b^
DPPH (%)	39.56 ± 0.41 ^b^	28.50 ± 0.34 ^a^

The results are presented as mean ± SD; different lowercase superscripts indicate Mann–Whitney U test significant differences (*p* < 0.05) within the same row.

**Table 7:** HPLC-DAD analysis of the samples.

Rt (λ)	Compound	Concentration (µg/g)			
		M	MD	Mulberry	Dandelion
3.810 (280)	Unidentified 1	8416.67 ± 255.41 ^a^	10,763.33 ± 361.43 ^b^	+	+
4.720 (280)	Unidentified 2	6033.33 ± 196.04	7163.33 ± 180.37	+	+
20.464 I 20.072 (360)	Neochlorogenic acid	+	+	1418.33 ± 213.62	575.07 ± 7.83
23.479 (360)	Caffeic acid	+	+	1778.33 ± 38.84	48.93 ± 0.53
23.889 (360)	Chlorogenic acid	+	+	398.10 ± 2.95	+
25.323 (360)	Quercetin glycoside 1	51.18 ± 1.81	50.47 ± 1.21	418.87 ± 17.11	66.03 ± 1.73
26.976 (360)	Riboflavin	73,290.00 ± 947.26 ^b^	60,252.33 ± 152.17 ^a^	nd	nd
28.475 (360)	Quercetin glycoside 2	63.33 ± 2.52 ^b^	54.90 ± 1.56 ^a^	+	+
30.054 (360)	Quercetin glycoside 3	65.09 ± 2.64	60.72 ± 1.56	+	+
30.677 (360)	Rutin	+	+	777.23 ± 7.53	3301.67 ± 7.64
31.268 (360)	Quercetin glycoside 4	43.79 ± 0.37 ^a^	50.35 ± 0.37 ^b^	844.60 ± 10.05	248.53 ± 1.40
32.217 (360)	Quercetin glycoside 5	+	+	590.83 ± 6.83	+
32.792 (360)	Quercetin glycoside 6	51.14 ± 0.22 ^b^	48.04 ± 0.79 ^a^	223.47 ± 1.47	+

Rt (λ): retention time of compounds; +—detected below quantification; nd—not detected; different lowercase superscripts indicate *t*-test (Unidentified 1, Unidentified 2, quercetin glycosides 1, 2, 3, and 6) or Mann–Whitney U test (riboflavin, quercetin glycoside 4) significant differences (*p* < 0.05) within the same row for larval powder samples.

## Data Availability

No datasets were created for this study apart from the results presented in the manuscript.

## Supplementary Materials

The following supporting information can be downloaded at: https://www.mdpi.com/article/10.3390/insects16020107/s1, Figure S1: Binocular view of silkworms feeding on dandelion leaves when presented with dandelion, lettuce, and radicchio; Figure S2: (a) Initial setup of a Petri dish with *B. mori* caterpillars in one corner and piles of chopped mulberry and dandelion leaves on opposite sides. (b) After 10 min, caterpillars are seen feeding, with some on the mulberry pile and two on the dandelion pile; Figure S3: Mature silkworms feeding in the mixed-diet group, clearly showing one individual eating a dandelion leaf despite the presence of mulberry leaves; Figure S4: Silkworms on a pile of mixed-diet leaves (dandelion and mulberry) chopped into strips.
