# Supplementary figures and images for: Diet Diversification in Bombyx mori Larvae: The Impact of Dandelion on Nutritional and Bioactive Profiles for Targeted Farming Goals
Source: Insects. 2025 Jan 22;16(2):107. doi: 10.3390/insects16020107 (PMC11855663; doi:10.3390/insects16020107)

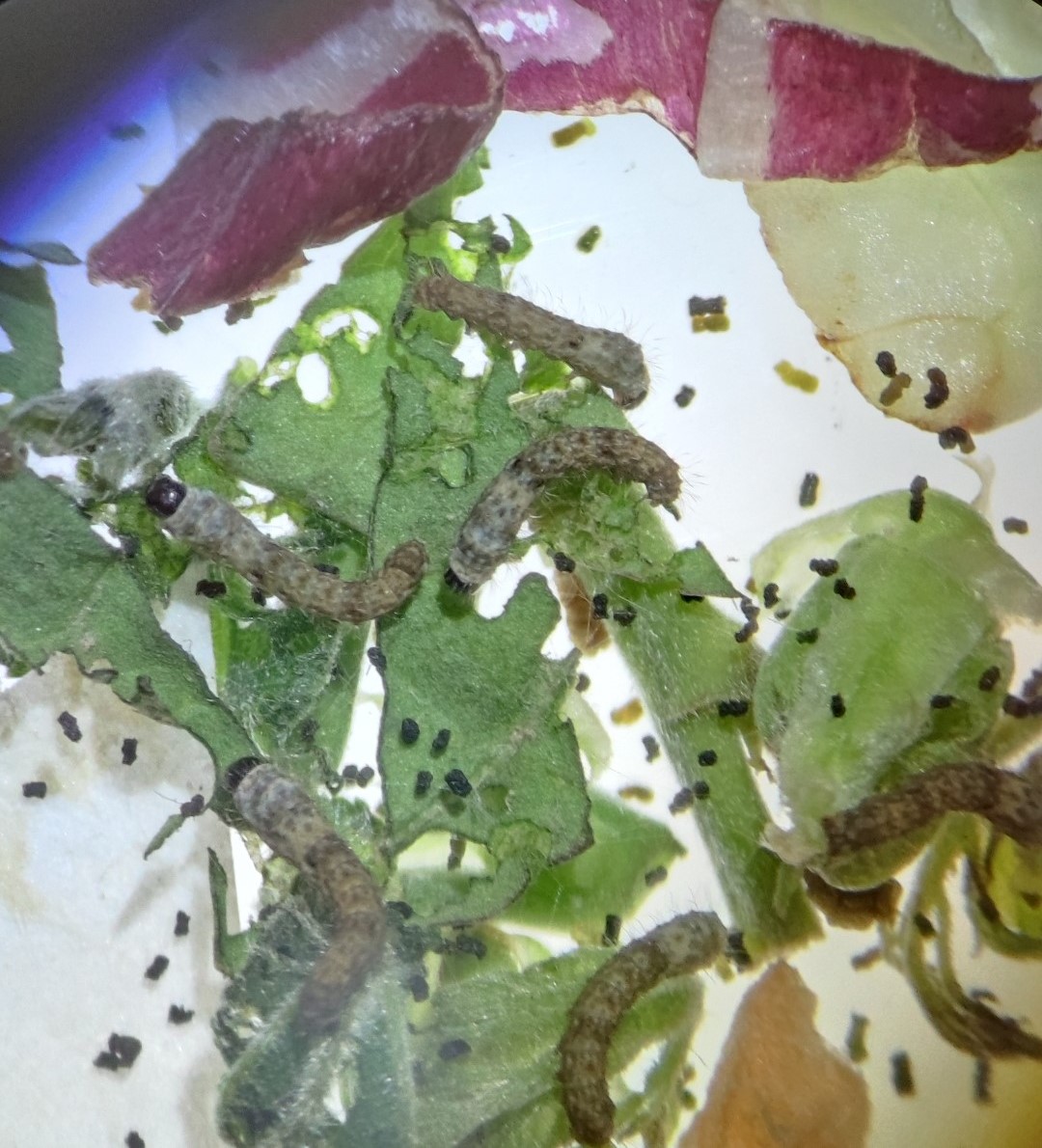

Supplement: Supplementary file 1 [file insects-16-00107-s001.zip › Figure S1.jpg]

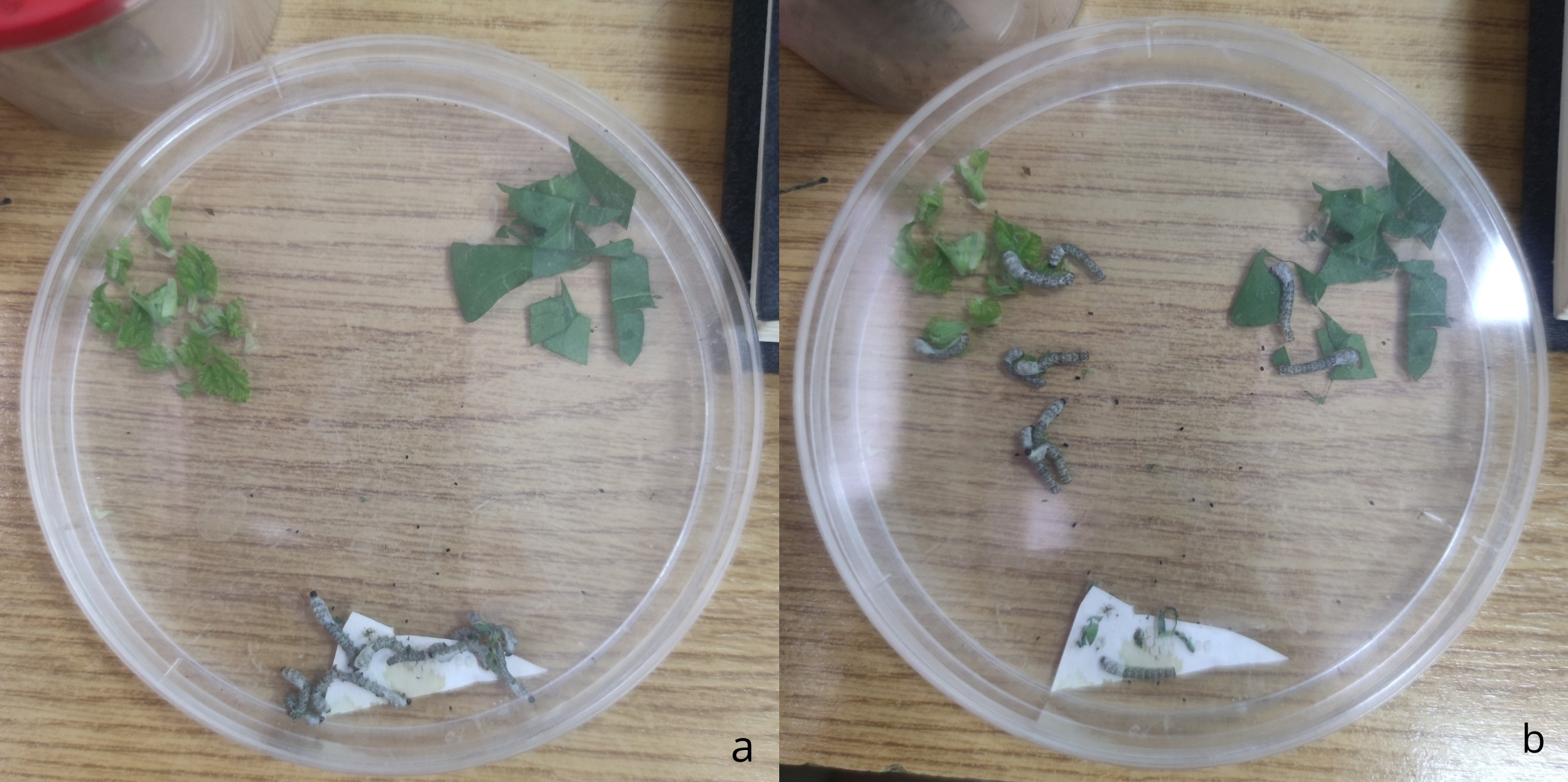

Supplement: Supplementary file 1 [file insects-16-00107-s001.zip › Figure S2.jpg]

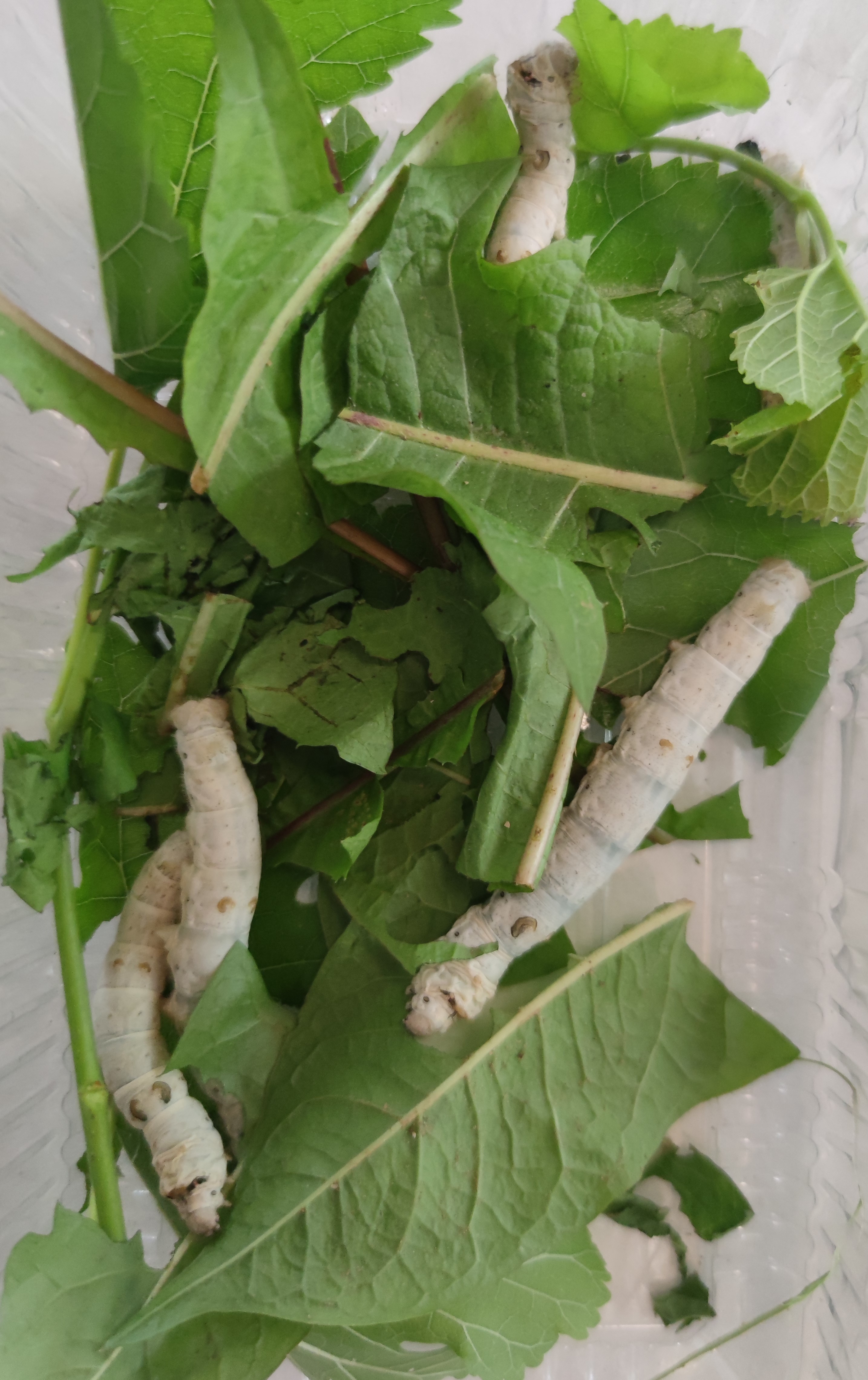

Supplement: Supplementary file 1 [file insects-16-00107-s001.zip › Figure S3.jpg]

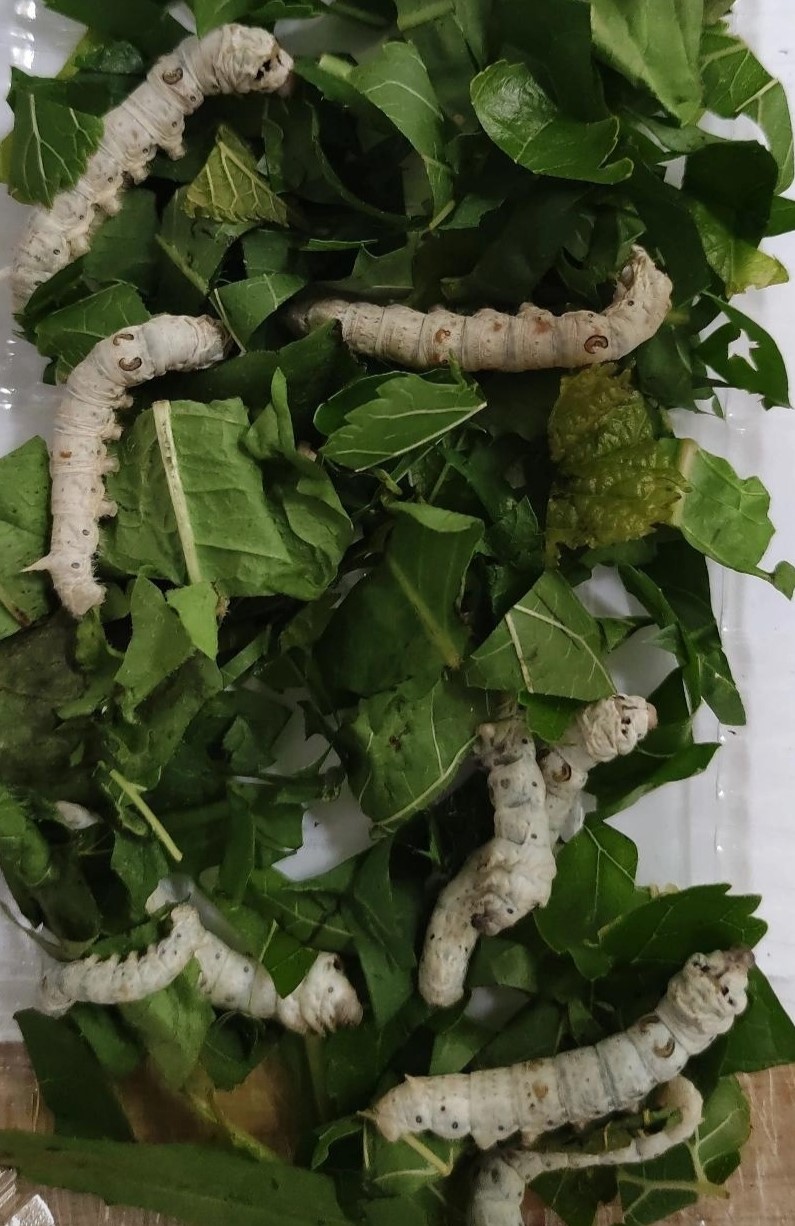

Supplement: Supplementary file 1 [file insects-16-00107-s001.zip › Figure S4.jpg]
